# Biophysical and computational insights from modeling human cortical pyramidal neurons

**DOI:** 10.3389/fnins.2025.1579715

**Published:** 2025-07-09

**Authors:** Sapir Shapira, Ido Aizenbud, Daniela Yoeli, Yoni Leibner, Huibert D. Mansvelder, Christiaan P. J. de Kock, Michael London, Idan Segev

**Affiliations:** ^1^The Edmond and Lily Safra center for Brain Sciences (ELSC), The Hebrew University of Jerusalem, Jerusalem, Israel; ^2^Department of Integrative Neurophysiology, Center for Neurogenomics and Cognitive Research (CNCR), Neuroscience Campus Amsterdam, VU Amsterdam, Amsterdam, Netherlands; ^3^Department of Neurobiology, The Hebrew University of Jerusalem, Jerusalem, Israel

**Keywords:** human neurons, pyramidal neurons, dendritic computation, compartmental modeling, biophysical modeling, machine learning models, single neuron computation, structure–function relationship

## Abstract

The human brain’s remarkable computational power enables parallel processing of vast information, integrating sensory inputs, memories, and emotions for rapid learning, adaptability, and creativity – far surpassing present-day artificial systems. These capabilities likely arise, in part, from the distinct properties of human neurons, which have only recently been elucidated through collaborative efforts among neurosurgeons, experimental, and theoretical neuroscientists. This effort has yielded unprecedented morphological and biophysical data on human neurons obtained during epilepsy or tumor surgeries. To integrate and interpret this diverse data, two complementary modeling approaches have emerged: detailed biophysical models, unraveling how morpho-electrical properties shape signal processing in human neurons, and machine learning models, which leverage the biophysical models to uncover hidden structure–function relationships. A major focus has been the disproportionately expanded layers 2/3 of the human cortex, where the large L2/3 pyramidal neurons (HL2/3 PNs) can track high-frequency input modulations, exhibit enhanced dendritic signaling, maintain numerous functional dendritic compartments, and display unique dendritic excitability. More recent efforts extend to modeling human hippocampal, cerebellar, and inhibitory cortical neurons. This review synthesizes key theoretical insights from biophysical and machine-learning models of HL2/3 PNs, and explores their implications for understanding “what makes us human.”

## 1 Introduction

The remarkable cognitive abilities of humans are often attributed to the expansion and specialization of the cerebral cortex, with its ∼16 billion neurons (Herculano-Houzel, 2009) and unique connectivity patterns (Seeman et al., 2018; Loomba et al., 2022; Campagnola et al., 2022; Hunt et al., 2023; Kanari et al., 2024; Peng et al., 2024). Of particular interest is whether, on top of this expansion, our remarkable cognitive abilities also rely on distinct properties of the building block of the cortex – the cortical neuron. An answer to this question requires appropriate tissue samples – a resource that has long been scarce. To overcome this, in the past decade, research teams around the world have collaborated with neurosurgeons to obtain tissue from surgeries performed to treat intractable epilepsy or tumors. As a result, direct characterization of the morphological, electrophysiological, synaptic, and genetic features of human neurons has become feasible (Eyal et al., 2014; Szegedi et al., 2016; Deitcher et al., 2017; Hodge et al., 2019; Berg et al., 2021; Hodge et al., 2020; Kalmbach et al., 2021; Chameh et al., 2023; Lee et al., 2023; Chartrand et al., 2023; Siletti et al., 2023).

These studies, together with recent electron-microscopic-based (“connectomics”) investigations (Loomba et al., 2022, Shapson-Coe et al., 2024) show that the disproportional expansion of the human cerebral cortex, and in particular that of the supragranular cortical layers 2 and 3 (DeFelipe, 2011; Galakhova et al., 2022), is accompanied by unique subcellular, cellular and network properties, including distinct transcriptomic profiles (Boldog et al., 2018; Berg et al., 2021; Chartrand et al., 2023), novel cell types (Deitcher et al., 2019; Berg et al., 2021; Mertens et al., 2024) human-specific synaptic connection (Lourenço and Bacci, 2017; Hunt et al., 2023; de Kock and Feldmeyer, 2023; Peng et al., 2024), an increased percentage of interneurons (Loomba et al., 2022), specialized dendritic morphology (Kalmbach et al., 2021; Masoli et al., 2024), and axonal ion channels (Gooch et al., 2022; Szegedi et al., 2023; Wilbers et al., 2023) and increases in dendritic compartmentalization (Beaulieu-Laroche et al., 2018; Eyal et al., 2018). Together, these distinctive features could potentially support our capacity for language, foresight and creativity, enabling us to create art, advance science, manipulate our own genes and brains, and build machines that can rival (and sometimes surpass) our own cognitive abilities (reviews in Galakhova et al., 2022).

What are the implications of these distinct features of human neurons for their computational capabilities? To address this question, we need neuron models that can systematically integrate diverse experimental data; such models have been developed in the last decade, yielding important biophysical computational insights at the single cell as well as the network levels. In this work we consolidate the insights at the neuron-level, in particular those gained from the majority of models that focused on the large and morphologically complex human L2/3 pyramidal neurons (HL2/3 PNs), but also from recent models of other types of human neurons. We then propose the next steps toward deepening our understanding of the input–output (I/O) relationship and computational capabilities of human (and other) neurons and how these properties might scale up to shape the organization and function of the networks they form.

**FIGURE 1:**
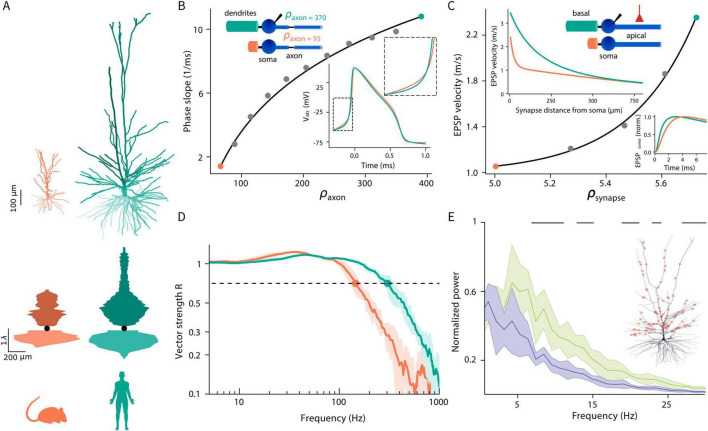
Biophysical insights gained from modeling human cortical pyramidal neurons. **(A)** Top: representative examples of mouse (orange) and human (green) L2/3 cortical pyramidal neurons. Bottom: equivalent cables as seen from the soma (black circle) for the cells shown on top, with basal/apical cables extending downward/upward, respectively. Due to the large surface area of the basal and oblique dendrites in humans, a large load is imposed on their soma and axon initial segment. **(B)** Human neurons display steep (“kinky”) axonal APs. Neuron models are composed of an infinite cylindrical axon with “hot” initial segment and passive cylindrical dendritic cables of variable length (variable load) coupled to the soma (two examples shown in top inset). The phase slope (steepness) of the axonal AP in these neuron models, recorded at the start of the modeled axon, is shown as a function of the load imposed on the axon initial segment (*ρ_*axon*_*) (gray circles). Orange (human) and green (mouse) circles correspond to *ρ_*axon*_* values computed from the detailed neuron models shown in panel **(A)**. Bottom inset depicts the axonal APs in the respective models. The zoom-in highlights the steeper AP “foot” in human (Adapted with permission from “Dendritic load, AP onset rapidness, and the tracking of high-frequency modulation by axonal spikes” by Guy Eyal, Huibert D. Mansvelder, Christiaan P. J. de Kock and Idan Segev, licensed under CC BY-NC-SA 3.0). **(C)** EPSP velocity is faster in human dendrites. Top inset shows the reduced models used, consisting of soma coupled to two passive cylinders: an apical cylinder receiving an excitatory synapse (red) and a basal cylinder of variable length (imposing variable load). Inset below shows EPSP velocity as a function of the distance of the synapses from the soma. For this case, ρ = 20 for the mouse and ρ = 40 for the human models; these values were computed from the detailed neuron models shown in panel **(A)**. Main curve shows EPSP velocity as a function of the load (*ρ_*synapse*_*) as for an exemplar synapse, located at *x*_*syn*_ = 50 μm from the soma. Orange and green circles correspond to the mouse and human neuron models, respectively. Right inset shows the soma EPSP in the human (green) and mouse (orange) models (Adapted with permission from “Impact of conductance load of the basal tree on excitatory postsynaptic potentials (EPSPs) velocity and latency” by Gáspár Oláh, Rajmund Lákovics, Sapir Shapira, Yoni Leibner, Attila Szücs, Éva Adrienn Csajbók, Pál Barzó, Gábor MolnárIdan Segev and Gábor Tamás licensed under CC BY 4.0). **(D)** Models of human neurons (green) effectively track high-frequency input modulations through their axonal spikes, as demonstrated by the respective shift-to-the-right of the vector strength as a function of input’s frequency modulations (Adapted with permission from “Dendritic load, AP onset rapidness, and the tracking of high-frequency modulation by axonal spikes” by Guy Eyal, Huibert D. Mansvelder, Christiaan P. J. de Kock and Idan Segev, licensed under CC BY-NC-SA 3.0). **(E)** h-Channels, which are prominent in human (but not in mouse) dendrites, facilitate the transfer of theta-frequency inputs from dendrites to the soma in human L3 pyramidal neurons. The power spectrum of the somatic membrane potential is shown for a modeled human pyramidal neuron (inset) stimulated by 1,000 synapses distributed along the apical dendrite. Green and blue traces indicate conditions with and without I_h_ channels, respectively. Black bars denote statistically significant differences in the power spectrum (Kolmogorov–Smirnov test; *p* < 0.01). Data is presented as mean ± SD (Adapted with permission from “Ih Affects the Subthreshold Integrative Properties of a Morphologically Precise Human L3 Pyramidal Neuron Model” by Kalmbach et al., licensed under CC-BY 4.0).

The first set of insights (Figure 1) provides model-based biophysical explanations for four experimental observations in human HL2/3 PNs: (i) the steep (“kinky”) somatic/axonal action potentials (APs) in these cells; (ii) the accelerated propagation speed of excitatory postsynaptic potentials (EPSPs) in dendrites of HL2/3 PNs; (iii) the ability of these cells to reliably track fast input modulations through axonal APs; and (iv) the effective transfer of theta frequencies from dendrite-to-soma in these cells.

**FIGURE 2:**
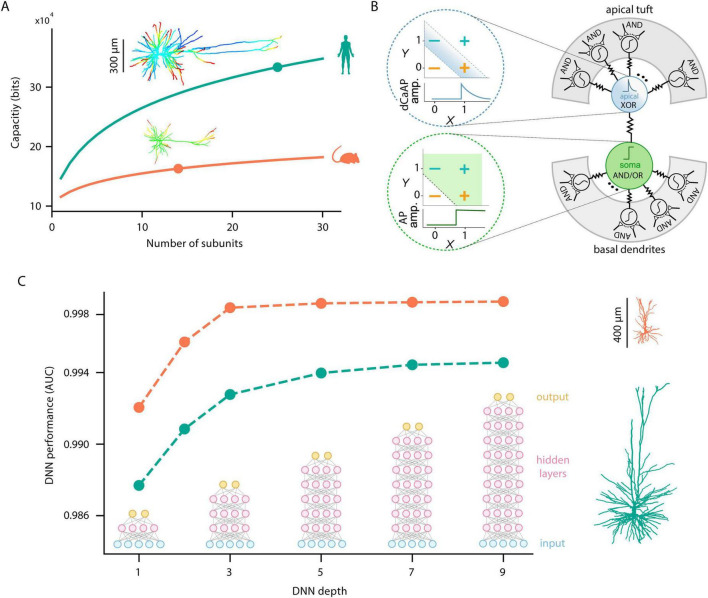
Computational insights from modeling human cortical pyramidal neurons. **(A)** Enhanced storage capacity of human L2/3 pyramidal neurons. Inset shows human (top) and mouse (bottom) modeled neurons in which the entire dendritic tree was activated with clusters of excitatory synaptic inputs. In HL2/3 PNs about 25 NMDA-spikes could be generated independently and simultaneously as compared to only 14 in rat L2/3 PNs. Graphs show the storage capacity as a function of the number of non-linear dendritic subunits per neuron, computed when the neurons were considered as a two-layer model as in Poirazi and Mel (2001) top and bottom curves are for 30,000 and 10,000 synaptic inputs, respectively (Adapted with permission from “Human L2/L3 pyramidal neurons have larger storage capacity compared to rat” by Guy Eyal, Matthijs B. Verhoog, Guilherme Testa-Silva, Yair Deitcher, Ruth Benavides-Piccione, Javier DeFelipe, Christiaan P. J. de Kock, Huibert D. Mansvelder and Idan Segev, licensed under CC BY 4.0). **(B)** HL2/3 PNs can implement distinct logical operations through its subcellular nonlinear compartments. The apical dendrite (top right, blue) produces dendritic calcium action potentials (dCaAPs) in response to either input pathway X or input pathway Y, but not when both pathways were active together, thus implementing a XOR-like operation (top left). In contrast, the somatic compartment (bottom right, green) implements OR-like computation, producing an action potential whenever one or more dendritic inputs reach threshold (bottom left). The basal and tuft dendrites (gray) function as AND gates via NMDA receptor-dependent spikes (Adapted with permission from “Anti-coincidence in L2/3 of the human cortex” by Albert Gidon, Timothy Adam Zolnik, Pawel Fidzinski, Felix Bolduan, Athanasia Papoutsi, Panayiota Poirazi, Martin Holtkamp, Imre Vida and Matthew Evan Larkum, licensed under CC BY 4.0). **(C)** Greater computational complexity of HL2/3 PNs using machine-learning (DNN) approach. Performance of Deep Neural Networks (DNNs) of various depths in replicating the input–output (I/O) function of detailed biophysical models of L2/3 PNs of human (green) and rat (orange) shown at right. As the depth of the DNN increases, the model’s performance (assessed by the AUC, Beniaguev et al., 2021) improves for both species. However, the AUC in human remains below that of rat, implying that the I/O function of the human model is more complex, consistently requiring deeper networks to approximate the respective I/O function (Adapted with permission from “Human cortical pyramidal neurons are more functionally complex compared to rat cortical pyramidal neurons” by Ido Aizenbud, Daniela Yoeli, David Beniaguev, Christiaan PJ de Kock, Michael London and Idan Segev licensed under CC BY-NC-ND 4.0).

The second set of insights (Figure 2) highlights the enhanced computational capabilities of HL2/3 PNs, showing that: (i) these neurons are highly compartmentalized, endowing them with large capacity for parallel processing and local nonlinear transformations – alongside multi-site plastic processes – prior to final integration in the axon; (ii) these cells can perform sophisticated computations, including XOR operation, through specialized nonlinear dendritic currents; and (iii) HL2/3 PNs exhibit a greater “depth” of I/O operations, as demonstrated by machine-learning approaches and analogs deep neural networks (DNNs) derived from their detailed biophysical models.

## 2 Biophysical insights from modeling HL2/3 PNs

### 2.1 Loaded with (human) potential: a unifying theoretical explanation for the enhanced signaling in HL2/3 PNs

A unifying theoretical explanation for the three experimental results shown in Figures 1A–C relies on the notion of “dendritic impedance load” (henceforth “dendritic load” or just “load”), imposed by the cable properties of the neuron on any particular dendritic/somatic/axonal site. This notion emerged from the pioneering work of W. Rall, where he solved the cable equation for dendritic neurons, showing that dendrites are electrically distributed cables rather than isopotential elements and that consequently, longitudinal (axial) current flows along the dendritic tree in response to local voltage perturbation (Rall, 1959; Rall, 1967; Rall, 1969; Rall, 1977). This work showed that the extensive membrane surface area of the dendrites “loads” the soma with additional capacitance and conductance, and that this affects both the magnitude and the temporal characteristics of the somatic excitatory synaptic potentials (EPSPs) originated in the dendrites (Rall, 1967; Rall, 1969; Rall and Rinzel, 1973; Rinzel and Rall, 1974).

Specifically, Rall (1959) solved the one-dimensional cable equation for the case of a cylindrical cable of finite length coupled to an isopotential soma (“ball and stick” model), showing that the voltage response, *V*(*t*), at any dendritic location (including the soma) to current injected at some location can be expressed as a sum of infinitely many exponential decays,


(1) $$V\,\left(t\right)=c_{0}\,e^{-\frac{t}{\tau_{0}}}+c_{1}\,e^{-\frac{t}{\tau_{1}}}+c_{2}\,e^{-\frac{t}{\tau_{2}}}+\ldots$$

where *τ_0_* = *τ_*m*_*, is the membrane time constant and *τ_0_* > *τ_1_* > *τ_2_*.

For a given dendritic tree, the coefficients *c_0_, c_1_, c_2_*, depend on the dendritic location *x* and the initial conditions over the tree, whereas the time constants, *τ_0_*, *τ_1_*, *τ_2_*, …, are independent of *x*. Rall referred to, *τ_1_*, *τ_2_*, …, as “equalizing time constants,” because they determine the rate at which voltage differences equilibrate between the perturbed dendritic site(s) receiving synaptic inputs and other dendritic regions (Equation 1). To assess the impact of the dendritic load (the load imposed by the cylindrical cable on the soma) on the equalizing time constant, Rall introduced the parameter ρ, the “dendrite-to-soma conductance ratio,”


(2) $$\rho =G_{dendrite}/G_{soma}$$

where *G*_*dendrite*_ is the input conductance of the cylindrical (dendritic) cable and *G*_*soma*_ is the input conductance of the soma (when Isolated). We will use below the term “load” to quantify the load imposed by the rest of the structure at the recorded compartment (i.e., the dendrites imposing their load on the soma or on the dendrites + soma imposing their load on the axon initial segment, etc.).

Rall demonstrated that the larger the dendritic load (the larger ρ is) the faster the voltage develops/decays at the soma in response to (synaptic or injected) current (Rall, 1969). It is worth noting that *G*_*dendrite*_ increases with increase in dendritic surface area and, therefore, ρ increases in neurons with large dendritic trees.

The load, ρ, in Equation 2 can be computed not only for a cylindrical dendrite but also for the general case, where the dendritic tree, as seen from the soma, is mathematically equivalent to a cable (or several cable emerging from the soma) with variable diameter, *d*_*eq*_(*X*),


(3) $$d_{e\,q}\,\left(X\right)={\left(\sum_{j}{\left(d_{j}\,\left(X\right)\right)}^{3/2}\right)}^{2/3}$$

where *X* is the cable (electrotonic) distance from the soma (in units of the electrotonic length, λ) and *d*_*j*_ is the diameter of the *j-*th dendrite at the distance *X* from the soma.

Typical equivalent cables, as seen from the soma, are shown in Figure 1A, bottom, for human (green) and mouse (orange) L2/3 PNs shown at Figure 1A, top. These cables start with a small diameter near the soma, which then increases with *X* as more dendritic branches emerge and then decreases with *X* as the number/area of distal dendrites decreases. In the cable shown in Figure 1A, bottom, Equation 3 was used to compute the equivalent cable separately for the apical and basal trees. As can be seen, the membrane surface area near the soma is much larger in human versus mouse L2/3 PNs, implying that ρ is larger in humans (see below).

We used Equation 2 to compute ρ for the two neurons shown in Figure 1A, assuming specific membrane resistance, *R*_*m*_ = 15,000 Ωcm^2^ and axial resistance, *R_*a*_* = 150 Ωcm, ρ = 19.6 for the mouse neuron and 40.2 for the human neuron. Consequently, the equalizing time constants are smaller in human L2/3 PNs and, in turn, the kinetics of voltage in the soma are expected to be faster (Eyal et al., 2014; Oláh et al., 2025).

The “somato-centric” load computed above can be extended for any dendritic (“dendro-centric”) or “axo-centric” viewpoints. The respective “equivalent cables” offer a graphical/analytical appreciation of the degree of load as “seen” from the viewpoint of any dendritic spine/synapse or axonal location. As shown below, these cables and their respective equalizing time constants, explain both the enhanced upstroke (or “foot”) of the AP in the axon’s initial segment as well as the increase of propagation speed of the EPSPs in human versus rodent’s dendrites. It also provides an analytical understanding of the dynamic of local dendritic voltage (e.g., in dendritic spines) in response to local synaptic inputs (Mertens et al., 2024; Sapir et al., unpublished data). These equivalent cables also provide useful insights into the degree of electrotonic decoupling between specific dendritic regions. Indeed, if the equivalent cable as seen from dendritic location #1, shows a large diameter change on the path to dendritic location #2, then these two dendritic locations are electrically decoupled (Leibner et al., unpublished data).

### 2.2 The “kinky” action potential enables HL2/3 PNs to track fast input modulations

In a series of innovative theoretical and experimental studies it was demonstrated that the encoding capability of the axonal APs to track fast-modulated synaptic input is primarily determined by the steepness (or “kinkiness”) of the AP upstroke (Naundorf et al., 2006; Baranauskas et al., 2010; Linaro et al., 2018; Ilin et al., 2013). The steeper the AP, the better the encoding. It was further found that HL2/3 PNs can reliably encode modulated inputs at frequencies of up to around 400–600 Hz, compared to only 100–200 Hz in rat L2/3 PNs (Testa-Silva et al., 2014) and that APs in HL2/3 PNs are indeed steeper than those in rats (Goriounova et al., 2018). These results raised two key questions: what causes the increased steepness of the APs in HL2/3 PNs and whether this on its own is sufficient to explain their superior ability to track rapid input modulations?

The dependency of the AP kinkiness on the neuron’s cable properties is best explained through Rall (1957) early theoretical work where he showed that, in an infinite passive cable, the voltage response, *V*(*t*), to a step current pulse reaches about 84% of its maximum after one membrane time constant, *τ_*m*_*. This is in contrast to an isopotential (spherical, R-C) neuron, where *V*(*t*) reaches only 63% of its maximum at *t* = *t*_*m*_. This is because, in an isopotential case, *V*(*t*) is governed solely by the (slow) membrane time constant, *τ_*m*_*, whereas in the infinite cable, the faster equalizing time constants discussed above enhance *V*(*t*) kinetics.

Because the upstroke (or “foot”) of the AP is primarily passive (Jack et al., 1975), it is strongly affected by the passive properties of the axonal cable. The AP is therefore expected to be steeper in the electrically distributed axon as compared to a “space clamped” (isopotential) axon, as indeed was demonstrated by Hodgkin and Huxley (1952). Importantly, this fundamental insight implies that there is a strong interaction between membrane excitability and neuron morphology in shaping the regenerative/spiking response of neurons (see also Mainen and Sejnowski, 1996).

Figure 1B (adapted from Eyal et al., 2014) shows the dependence of the steepness of the AP (its phase slope, see definition in Eyal et al., 2014) on the load imposed on the axon initial segment by the dendrites/soma. This load is termed here as *ρ_*axon*_* (Hay et al., 2013). Neuron models are composed of an isopotential soma coupled to an infinite cylindrical axon with “hot” initial segment (containing high-density voltage-gated Na^+^ ion channels) and passive cylindrical dendritic cables of variable length (imposing variable load, *ρ_*axon*_*, on the axon initial segment). Examples for two such models are shown in Figure 1B, top inset. Orange (human) and green (mouse) circles correspond to *ρ_*axon*_* values computed for the detailed neuron models shown in Figure 1A. Gray circles denotes different *ρ_*axon*_*, bottom inset in Figure 1B depicts the APs at the start of the axon’s initial segment in the respective human (green) and mouse (orange) models. The zoom-in highlights the steeper “foot” (or increased “kinkiness”) of the AP in human.

Because of the particularly large/extensive basal and oblique trees in HL2/3 PNs, *ρ_*axon*_* is large in these cells and, consequently, the equalizing time constants are small in their soma/axon initial segment (Hay et al., 2013; and see mathematical explanations in Eyal et al., 2014). In turn, the AP is kinkier in these cells and, as Eyal et al. (2014) have showed, this, in itself, explains the more reliable tracking of high-frequency input modulations by axonal spikes (thus, the enhanced encoding capabilities in HL2/3 PNs; Figure 1D). It is interesting to note that this increase in AP steepness is expected to improve network synchrony in human cortical circuits (Ilin et al., 2013). Human ion channel properties and Na^+^ channel availability was shown to serve as an additional mechanism that supports fast AP signaling in HL2/3 PNs (Wilbers et al., 2023).

### 2.3 EPSPs propagation speed is accelerated in dendrites of HL2/3 PNs

It was recently found that EPSPs travel approximately 30% faster in the apical dendritic tree of HL2/3 PNs compared to rats’ L2/3 PNs (0.9 m/s versus 0.7 m/s, on average, respectively; Oláh et al., 2025). This acceleration helps to compensate for the larger signal delay expected due to the greater soma-to-soma path-length in human cortex (axon + dendrite path length is 385 ± 74 μm in human and 262 ± 53 μm in rat). Whereas part of this EPSPs acceleration is attributed to the larger diameter of the apical dendrites in human neuron**s**, detailed compartmental models show that diameter differences alone account for only a small fraction of the increase in EPSP speed (Oláh et al., 2025). Instead, these models show that the primary mechanism behind the faster EPSP propagation is the shorter equalizing time constants in HL2/3 PNs, resulting from the large load imposed by the basal dendritic tree on the apical dendrite in these cells (Figure 1C, adapted from Oláh et al., 2025).

The top inset in Figure 1C shows the simplified models used to explore the enhanced EPSP velocity in HL2/3 PNs. These models consist of soma coupled to a passive apical cylinder, receiving an excitatory synapse (red), and a passive basal cylinder of variable length (imposing variable load, ρ, on the apical tree). Inset below shows EPSP velocity as a function of the distance of the synapses from the soma for the case where ρ = 20 (mouse model, orange) and ρ = 40 (human model, green). These values were computed from the detailed neuron models shown in Figure 1A. The main curve shows EPSP velocity as a function of the load as “seen” from the synapse (*ρ_*synapse*_*) that is located at *x*_*syn*_ = 50 μm from the soma. Orange and green circles correspond to the mouse and human neuron models, respectively. Right inset depicts the zoom-in EPSP at the soma, showing that it reaches the soma earlier in human versus mouse models.

To confirm that EPSP acceleration in the human apical tree is indeed a consequence of the large load imposed by the basal tree, we constructed “hybrid neuron models” in which the basal tree of human HL2/3 PNs was replaced with that of a rat. In these hybrid models, the enhanced EPSP speed in human apical dendrites diminished. Conversely, when the rat’s basal tree was replaced with that of a human, EPSP propagation speed increased in the apical tree of the rat’s L2/3 PNs. Notably, the faster EPSP propagation in HL2/3 PNs is expected to occur not only in the apical tree but also in the basal dendrites. This is because, in humans, each basal branch experiences a large load imposed by all other basal branches combined, as well as by the apical tree (Oláh et al., 2025). Note that the same principles apply equally to rat neurons, only there, the load imposed by all other basal branches on a particular basal dendrite is smaller compared to that of human.

### 2.4 Prominent h-channels in HL2/3 PNs promote the transfer of theta frequencies from dendrite-to-soma

Using combined experimental and detailed compartmental modeling, Kalmbach et al. (2018) investigated how the stronger h-channel-related membrane properties in HL2/3 PNs compared to mouse contribute to differences in rodent versus human neuronal physiology. They applied synaptic inputs, distributed along the apical dendrite, in a modeled HL2/3 PNs, both with and without h-channels (Figure 1E). They showed that h-channels modulate subthreshold membrane potential dynamics and firing patterns, and pinpointed the role of these channels in promoting the transfer of theta frequencies from dendrite-to-soma in these neurons. They suggest that this might contribute to the dominant delta/theta band oscillations apparent in the human supragranular cortex (Halgren et al., 2018) and to memory-related theta-frequency phase-locking of single human neurons observed *in vivo* (Jacobs et al., 2007; Rutishauser et al., 2010). Kalmbach et al. (2018) showed that *I*_*h*_ produced EPSPs at the soma of the model L3 human PN with a significantly faster time course, thus reducing the temporal summation of synaptic inputs at the soma. By opposing changes to membrane potential, *I*_*h*_ induces phenomenological inductance to the neuron’s membrane. This counteracts signal delay imposed by membrane capacitance, promoting the transfer to the soma of synaptic input containing theta frequencies.

Kalmbach et al. (2018) study also suggests that the prominent h-channels in HL2/3 PNs may significantly affect the spike initiation dynamics in these cells and can switch the firing mode of a neuron from temporal integrator to coincidence detector, whereby spiking is sensitive to correlated synaptic input rather than changes in mean presynaptic firing rate. Additional work supporting the distinct role of h-channels (“sag voltage”) in human cortical neurons can be found in Chameh et al. (2021), Berg et al. (2021), and Guet-McCreight et al. (2023).

## 3 Computational insights from modeling HL2/3 PNs

### 3.1 HL2/3 PNs have increased number of functional dendritic compartments

Rall’s cable theory for dendritic trees has demonstrated that, because of cable filtering, different parts of the dendritic tree are electrically decoupled from each other and, consequently, they could act as semi-independent compartments (Rall, 1959, Rall and Rinzel, 1973; Rinzel and Rall, 1974). In each compartment, local synaptic inputs may be integrated nonlinearly and undergo synaptic plasticity before they impact the neuron’s overall output (Segev and Rall, 1998; Koch and Segev, 2000; London and Häusser, 2005; Losonczy et al., 2008; Makara et al., 2009; Cichon and Gan, 2015; Otor et al., 2022).

Modeling studies have shown that dendritic compartmentalization combined with non-linear properties (e.g., of the synaptic input itself or the dendritic membrane, or both) endows the neuron with the capability to perform multi-site (AND-NOT type and XOR) logical operations (Koch et al., 1982; Shepherd and Brayton, 1987; Gidon et al., 2020), improved temporal coincidence detection (Agmon-Snir et al., 1998), perform nonlinear pattern discrimination (Mel, 1992, Moldwin et al., 2021; Beniaguev et al., 2024), enabling neurons to self-organize to solve a binding problem (Legenstein and Maass, 2011) and providing, via “dendritic gating,” flexible routing of information flow in a complex brain network (Yang et al., 2016). Indeed, dendritic mechanisms have inspired innovative solutions for significant AI-related problems, including credit assignment in multi-layer networks, catastrophic forgetting, and very high-power consumption of present-day AI learning algorithms (Pagkalos et al., 2024).

“Dendritic compartments” can be defined in multiple ways. One approach is based on calculating the transfer resistance (*R*_*i,j*_) between dendritic locations; if *R*_*i,j*_ is low, the locations are electrically decoupled and belong to separate compartments (Koch et al., 1982; Eyal et al., 2018; Dan et al., 2018). Another method, proposed by Rabinowitch and Segev (2006), defines compartments as sub-regions where background synaptic activity is similar but distinct from other areas. They identified compartments using cross-correlations between membrane potential traces at different dendritic sites.

A more direct measure of the number of “functional compartments” in a dendritic tree was proposed by Eyal et al. (2018). They calculated how many independent (electrically isolated) NMDA spikes could be generated simultaneously in a modeled neuron without triggering an axonal spike. Their findings showed that, due to the greater number of dendritic branches, extended cable length, and especially the abundance of oblique dendrites in HL2/3 PNs, these neurons could generate about 25 independent NMDA spikes simultaneously, compared to only 14 in rat L2/3 PNs (Figure 2A). Notably, the number and size of these dendritic compartments are dynamic and can be modulated by factors such as synaptic inhibition and branch excitability (Gidon and Segev, 2012; Wybo et al., 2019).

Using Poirazi and Mel (2001) formulation for computing the memory capacity of a neuron taking into account both the number of independent compartments and the number of synapses, HL2/3 PNs show 10-folds increase in memory compared to rodents L2/3 PNs (Figure 2A). A similar result for human versus rat CA1 PNs was recently obtained by Mertens et al. (2024). Furthermore, by combining modeling and direct electrical recordings from dendrites, Beaulieu-Laroche et al. (2018) found enhanced electrical compartmentalization also in human L5 PNs.

### 3.2 Unique nonlinear dendritic current enables XOR operation in HL2/3

In a recent study, Gidon et al. (2020) used dual recordings from the soma and apical dendrite of HL2/3 PNs. They discovered a new class of calcium-mediated dendritic action potentials (dCaAPs) whose waveform and effects on neuronal output have not been previously described. These dendritic action potentials were found to be graded – their amplitudes were maximal for threshold-level stimuli but dampened for stronger stimuli. They then used a detailed compartmental model of a HL2/3 PN and simulated the behavior of dCaAP including its threshold, width, and amplitude as a function of the input strength to investigate the functional outcome of the dCaAP activation function.

Using their model, Gidon et al. (2020) showed that the apical dendrites of HL2/3 PNs, through localized dCaAPs, can implement an XOR operation – responding to either of two excitatory inputs but not both simultaneously (Figure 2B, blue). In contrast, the soma performs an OR-like operation, firing an AP when one or more inputs are present (Figure 2B, green), whereas the basal and tuft dendrites act as AND gates via NMDA receptor-dependent spikes (Figure 2B, gray). The presence of dCaAPs in HL2/3 PNs, enabling natural XOR computations, gives human L2/3 neurons multi-layer-like network capabilities – surpassing rat L2/3 PNs, which lack this unique nonlinear membrane current.

### 3.3 HL2/3 PNs are computationally complex as assessed by their analogous DNNs

In a recent study, Aizenbud et al. (2024) proposed a novel method to gauge the functional complexity of single neurons by measuring how difficult it is for a DNN to replicate the neuron’s I/O dynamics. By training a temporal convolutional network of fixed width and depth to approximate both the subthreshold somatic voltage and spike outputs of the respective biophysically detailed models, they quantify the functional complexity arising from the neuron’s morphology and nonlinear synaptic properties.

A key observation is that human cortical PNs, particularly HL2/3 PNs, consistently present a greater challenge for the DNN to replicate the respective I/O function compared to the respective rat PNs. Aizenbud et al. (2024) show that because of the larger dendritic surface area and more complex dendritic tree, and because of the steeper NMDA-dependent nonlinearities (Eyal et al., 2018) the I/O response pattern is more complex in human PNs, requiring a deeper or more sophisticated DNN to replicate their I/O relationship.

Figure 2C compares the DNN performance, as measured by the area under the curve (AUC, Beniaguev et al., 2021), as a function of network depths for two representative neuron models L2/3 PNs from human and rat. For a fixed width of 128 nodes per layer and for every architecture configuration, the DNN models of human neurons yield lower AUC, indicating lower predictive accuracy and underscoring their elevated complexity relative to their rat counterparts. As discussed by Beniaguev et al. (2021), the depth of the DNN required for the modeling of a given neuron can be used (under certain assumptions) as an index for its computational power; the deeper it is, the more sophisticated computations this neuron could perform.

## 4 Discussion

In recent years there is accumulating evidence that numerous cellular and network features of the human cortex contribute to our advanced cognitive capabilities. But how could these diverse features be combined and linked together to explain our sophisticated cognitive capabilities? One approach is to use computational models that enable to progressively integrate these features and then use the model to explore how each additional feature incrementally enhances the model’s ability to replicate human-like cognitive functions.

Under this conceptual framework, this review synthesizes the key computational properties of human cortical neurons, derived from detailed experimentally grounded compartmental models and complementary machine-learning approaches. Focusing on the large and elaborated L2/3 pyramidal neurons (HL2/3 PNs) in the significantly expanded human cortical layer, this review summarizes the key insights gained from fine-scale models of these cells. It offers rigorous mathematical explanations for several experimental observations unique to these cells. It demonstrates how the morpho-electrical complexity of HL2/3 PNs boosts both their computational and memory capacity, thereby likely contributing to the enhanced cognitive capabilities of the human cortex as a whole.

Specifically, we provide a unified mechanism for explaining several key phenomena in human HL2/3 PNs, based on the notion of “impedance load.” We show that the large load imposed on the axon’s initial segment by the large dendritic tree makes the somatic APs “kinky” in these cells, and this underlies their capability to reliably track rapid input modulations. The load imposed on the stem dendrites emerging from the soma explains the enhanced speed of EPSP propagation in human dendrites; it also partially explains the extensive dendritic compartmentalization found in HL23 PNs, supporting enhanced, multi-site nonlinear parallel information processing within a single human neuron. Large dendritic trees are found in cortical neurons of other mammalian species as well. However, in human L2/3 PNs this extensive morphology operates in conjunction with distinctive ion channel properties and synaptic connectivity that, together, may uniquely enhance the computational repertoire of human cortical neurons, beyond what size alone accounts for.

Indeed, the review also highlights experimental findings and insights obtained from models regarding the computational impact of distinctive voltage-gated dendritic ion channels found in HL2/3 PNs: the high density of dendritic h-channels that shapes the resonance properties and improve dendrite-to-soma signal transfer (Kalmbach et al., 2018), the unique dendritic Ca^2+^ current that enables XOR-like operations in these cells (Gidon et al., 2020), and the steep voltage-dependence of NMDA-receptors, underlying NMDA-driven nonlinear activity as well as NMDA spikes [although, as noted by Testa-Silva et al. (2022), it remains uncertain whether NMDA spikes occur in all human neurons]. Aizenbud et al. (2024) showed using machine learning (DNN) approach that, when combined with the large and complex dendritic tree of HL2/3 PNs, the distinct nonlinear membrane/synaptic characteristics in human cells augment the computational capabilities of these neurons.

Whereas this review focuses on biophysical and computational insights specifically gained from models of human layer 2/3 pyramidal neurons (HL2/3 PNs), an important related question is the extent to which these insights can be generalized to other neuron types within the human cortex and subcortical regions. In particular, it remains to be explored whether the morphological complexity and specialized ion channel distributions observed in HL2/3 PNs are also present – and if so, whether they serve comparable roles – in other neuron subtypes, each of which contributes to the rich computational diversity of the human brain. Recent experimentally based models of other human neuron types have begun to address this question, revealing distinct morphological and electrical properties in hippocampal CA1 PNs (Mertens et al., 2024), cerebellar Purkinje cells (Masoli et al., 2024), and cortical inhibitory neurons (Boldog et al., 2018; Chartrand et al., 2023). These findings add to the growing body of evidence suggesting that human neurons – the brain’s fundamental computational “microchips” – are indeed more complex than their rodent counterparts.

The next major advancement in single-neuron modeling will come from integrating recent data obtained from dense, EM-based reconstructions of entire human cortical neurons (Loomba et al., 2022; Shapson-Coe et al., 2024). These high-resolution reconstructions provide unprecedented insights into the location and strength of excitatory and inhibitory dendritic synapses, as inferred from their physical size (Holler et al., 2021). Additionally, *ex vivo* measurements of individual synapses in human tissue – leveraging, for example, next-generation genetically encoded voltage indicators (GEVIs; Cornejo et al., 2022; Hao et al., 2024) – will significantly enhance both biophysical models and AI-based DNN models of single neurons. Consequently, these improvements will extend to network-level models as well. These powerful advances will refine our understanding of the parameters that shape the I/O properties and computational functions of cortical (and other) neurons in general, with a particular emphasis on human cortical neurons.

Noteworthy is that improvements of our understanding of the rich-set of computations performed at the single-neuron level will eventually extend to network-level models. Incorporating morphological and biophysical details into network simulations (Hay and Segev, 2015; Reimann et al., 2013; Dura-Bernal et al., 2024) as well as to DNN models (Egrioglu and Bas, 2024; Tang et al., 2024; Chavlis and Poirazi, 2025), will advance our understanding of the way local dendritic computations scale up to circuit-level dynamics and improve network computations.

As present-day EM data typically consist of relatively small volumes (and in many cases highly truncated morphologies) future neuron models will benefit from new imaging techniques capable of mapping larger volumes of human brain tissue than is currently feasible with traditional EM (Loomba et al., 2022; Shapson-Coe et al., 2024). For instance, expansion microscopy enables visualization of sub-diffraction-limit structures on a larger spatial scale (Balcioglu et al., 2023), while X-ray nano-tomography offers complementary insights at high resolution (Rodrigues et al., 2021; Bosch et al., 2024). When combined with whole-brain morphometric approaches (Liu et al., 2024), these methods provide a multi-scale perspective on neural architecture – from individual synapses to macro-level connectivity – including not only cortical areas but also hippocampal, cerebellar, and other subcortical structures.

By generating increasingly detailed maps of synaptic distribution and size, dendritic morphology, and circuit organization, this next generation of imaging will supply computational models with richer structural parameters. This, in turn, will allow us to explore, for example, via the simulation pipeline offered by the Open Brain Institute [OBI] (2025), how variations in anatomy and physiology contribute to human-specific information processing and the emergence of neurological and neuropsychiatric diseases (Rich et al., 2022; Dura-Bernal et al., 2024; Guet-McCreight et al., 2024), ultimately shedding light on “what makes us human.”
